# The gender factor in meeting the needs of women who use drugs in Senegal

**DOI:** 10.1186/s12954-025-01186-z

**Published:** 2025-04-11

**Authors:** Rose André Y. Faye, Alice Desclaux, Aïda Diagne

**Affiliations:** 1 IRD, TransVIHMI (IRD, INSERM, Université de Montpellier), Montpellier, France; 2 Centre régional de recherche et de formation à la prise en charge de Fann à Dakar, Dakar, Sénégal; 3 “Femmes engagées” Community-Based Organization, Dakar, Sénégal

**Keywords:** Women, Drugs, Addiction, Gender, Senegal, West Africa

## Abstract

Harm reduction (HR) services are developing in Africa. In Senegal, the overall HR service coverage ratio is one WUD for every 10 men. By analysing gender-specific initiatives developed by HR stakeholders and evaluating their limitations, we explored HR in Senegal through a gender lens to better understand how to meet the specific needs of WWUD. The data for this study were collected through interviews, observations, and the researchers’ direct presence in drug use settings, as part of a social anthropology research project on the trajectories of WWUD in Senegal. The study reveals that in Senegal, two approaches (broadly integrating HR) are employed to address the specific health and psychosocial needs of WWUD: care exclusively dedicated to WWUD (e.g., gynaecological consultations, women-only days, HIV testing, discussion groups) and support focused on capacity building (e.g., community empowerment, training peer workers). However, this dual approach has limitations. It primarily targets WWUD who inject drugs such as cocaine and heroin, neglecting the needs of those using non-injectable substances (e.g., tramadol, alcohol, cannabis). Additionally, it does not account for the diverse social profiles of WWUD and their varying needs. Despite HR actors’ best efforts to provide tailored services, they face funding challenges. Moreover, the stigma surrounding drug use hinders WWUD’s participation in proposed activities. To address the limitations and challenges of the current HR offer, WWUD employ adaptive and resilience strategies through community empowerment initiatives. Addressing these gaps requires the development of HR services tailored to the specific needs of non-injecting WWUD, conducting in-depth research to better adapt interventions for this target population, and mobilising funding to implement innovative and effective approaches to improve their access to care.

## Introduction

The first harm reduction (HR) programmes in Africa were implemented in the South and East of the continent in 2013. Although women who use drugs (WWUD) in Africa face specific barriers when attempting to access health services (i.e., HR and other care) due to various psychosocial and contextual factors, few studies to date have documented these barriers. They identified psychosocial barriers to treatment such as fear of stigma, shame, and WWUD’s lack of trust in healthcare professionals [1]. They also highlighted contextual obstacles linked to structural inequalities at various levels, such as the absence of the gender factor in HR programme agendas, the lack of childcare services, the lack of means of transport to travel to health structures, and poverty [1–4].

In West Africa, and particularly in Senegal, data on the characteristics of WWUD and how their specific needs are taken into consideration in HR programmes are relatively scarce [5]. Nonetheless, together with empirical observations, these data highlight that (1) WWUD’s HR experiences differs from that of men because they are fewer in number, and consequently, their specific needs are not considered; (2) they face greater gender-related stigma than men, partly because of Senegal’s sociocultural context, and (3) they are often systematically described by researchers as sex workers [6, 7].

In the context of HR in Senegal, the Integrated Addictions Management Centre in Dakar (CEPIAD) opened in 2014 with a mission to address the wide-ranging needs of drug users. It was the first such centre in French-speaking West Africa. Acting as a reference model, other similar structures have been created throughout the country in the last ten years. However, WWUD still only account for 10% of patients followed at CEPIAD. This inequity suggests that any study examining the limitations of current non-gendered and masculine HR policy paradigms in terms of WWUDs’ specific needs would need to use gender as an interpretative framework. Gender refers to a social construction of what is masculine and feminine. These aspects are often reduced to a male-female binarism reflecting the dominant social norms assigned to each of these groups [8]. These representations are historically linked to power relations and are advantageous to men. Social sciences study the question of gender using various approaches [9, 10]. The interpretation of gender that we propose in the present article does not consist of considering gender as a theme but as a distinct model of social and cultural differentiation. It is in no way a question of only considering women as a subpopulation or as a study object; rather it involves examining the social relations and power relations which influence perceptions of practices and problems linked to drugs in the context of women. The challenge is to highlight “gendered subjectivations” and lived experiences [11] in connection with the HR initiatives provided in the field of drug policy in Senegal.

Gender as an interpretive framework makes it possible to understand and analyse the invisibilisation of women in the HR programmes for people who use drugs (PWUD) in Senegal. Moreover, in the field of drug policy, the development of initiatives to respond to PWUD needs should take into account the gender factor by focusing on the specificities of men, women, queer and transgender people. However, available data on the condition of WWUD around the world show that they are often an invisible and minority group in HR programmes, and that they are described based on gender stereotypes (i.e., mothers, prostitutes) [12]. Added to this is the fact that the cultural model of drug addiction is considered in a non-gendered way (and is therefore defined on a masculine basis). This raises the need for more adapted and more equitable HR initiatives to correct the inequality generated by the “invisibilization” of women [13].

However, a gender-based approach to tackling drug addiction must take into account the biological and social aspects, experiences, and socially constructed identities of girls, women, boys, and men in a given society, and the influence of these different elements on drug-related problems [14]. “Gender-sensitive” HR services targeting WWUD are based on creating an environment that encourages the use of these services by this specific population. This implies that choosing where support will be given, staff selection, and the development of the services to be offered, must all reflect an understanding of WWUD’s specific needs [15].

We have talked about the need to use gender as an interpretative framework for any investigation of the limitations of HR policies and interventions in terms of WWUD’s needs. Just as in the field of maternal health, and more broadly in the fields of public health and development, as well as global health [16], HR interventions are standardized and developed by competent, technically relevant experts from international organizations, bilateral collaborations and non-governmental organizations (NGOs). These standardized interventions, which are based on changes in the behaviour of one or more categories of stakeholder (e.g., communities, key populations), using a “mechanism” and “systems” independently of social contexts, have been described as “traveling models” (also called “mobile models”, “circulating models”, “global templates” or “policy transfers”) [17]. Traveling models are frequently applied by health institutions in different African countries. However, in-depth field analyses, particularly by anthropologists, have shown that (1) interventions based on these models may be unsuitable for real-world health facilities, (2) models are often only partly implemented at the field level, and (3) they are disconnected from the local context [17]. Applying the logic of traveling models to gender-sensitive HR policies highlights how global interventions introduced in Senegal by international experts and funded by global programmes, are translated into local practices. Just as for the inequity highlighted above, applying the traveling models concept also underlines the need to integrate gender in any analytical framework in order to better understand the gaps between universal models and real-world context-specific needs.

The difficulties of implementing HR policies in Africa, particularly for women, are poorly documented. Given the context described in the different paragraphs above, this ethnographic study aimed to analyse the integration of gender into harm reduction initiatives in Senegal to better address the needs of women who use drugs. To achieve the study’s objective, we explored three key questions: (1) How do stakeholders perceive the vulnerability of women to drug use compared to men? (2) To what extent do existing initiatives integrate the gender dimension, and what are their limitations? (3) What strategies do women who use drugs adopt to meet their specific needs?

## Methodology

The qualitative study we describe here was based on data collected for a PhD thesis social anthropology of this article on the trajectories of WWUD in Senegal [5]. This thesis is associated with two Senegalese HR implementation and research projects, CODISEN and CODISOCS^1^, which the authors of this article participated in. From 2016 to 2019, CODISEN supported CEPIAD (see above), most of its input focusing on developing a model of care and prevention for people who inject drugs (PWIDs) in Dakar by integrating clinical, addiction-based and socio-anthropological aspects. As an extension to this work, since 2018, CODISOCS has explored the social dynamics induced by CEPIAD’s care system.

The study combined observations, individual interviews and focus groups from both projects. Specifically, between 2017 and 2020, during a period of immersion at CEPIAD and in places where drugs are consumed in Dakar and Mbour, we collected data through (1) the life narratives of 34 WWUD, (2) the life narratives of 10 male PWUD who did not attend CEPIAD or who dropped out of its treatment program after starting it, and (3) 10 interviews with actors in the field (care providers, field-based stakeholders, and HR specialists). As most ethnographic studies on the Senegalese drug-user population to date have focused on men, and knowledge about WWUD is limited, we adopted a feminist perspective. We chose to interview the 10 male PWUD in order to better understand the gendered barriers to accessing HR services.

To analyse complex social processes, a progressive data collection approach was adopted through repeated visits to various places (CEPIAD, living spaces (e.g., houses, other places to use drugs), shanty towns, nightlife settings). Meetings with WWUD in places where WWUD consumed drugs (were facilitated by peer workers. More specifically, peer workers encouraged peers to trust the researcher and convinced them about the confidentiality of the research study. A snowball sampling method, involving associations of sex workers, helped diversify the profiles encountered. Respondents included WWUD on addiction treatment for at least a month and WWUD with no access to care.

A life narrative helps an individual makes sense of his/her experience through an in-depth biographical individual interview. Life narratives were especially important in our ethnographic study. All interview data were anonymized. We audio-recorded all interviews with the consent of the participants. Data were translated from Wolof into French when necessary. We transcribed, recorded, and cleaned all the data. Data cleaning helped strengthen anonymization by using a pseudonym for each participant. We coded the interviews using the qualitative data processing software package Dedoose^2^. Data were analysed by comparison and triangulation using an inductive and iterative approach. We carried out fieldwork to evaluate the provisional partial interpretations and to complement data when necessary. From an ethical point of view, in addition to receiving official approval^3^, we were attentive to the needs expressed by participants. Specifically, she informed them about the services offered by CEPIAD and the Fann Regional Centre for Research and Training for Care (CRCF) with a view to searching for options for help within the current healthcare system, particularly for needs related to physical and mental health. Confidentiality was always respected from data collection and treatment right through to stakeholder communication and publication of the results.

## Study results

The study results are presented in two Sect. (3.1 and 3.2); the first concerns the suitability of HR and care for PWUD (and specifically for WWUD); the second concerns the gender factor in the Senegalese healthcare system in terms of achievements made and limitations.

### The suitability of current harm reduction and care for PWUD and specifically for WWUD

Before describing the way in which HR services respond adequately (or not) to the specific needs of WWUD, we must describe these needs and the situations experienced by these women, through a comparison with men.

#### Different situations, care utilisation, and needs for women and for men

Life narratives highlighted that WWUD were affected by different types of vulnerabilities associated with sex work, the relationship with their partner, stigmatization, and motherhood. These vulnerabilities underpinned their life and drug use trajectories and influenced their drug consumption and related care utilization. In some life narratives, sex work trajectories intersected with those of drug use.

The vulnerabilities described by the WWUD were linked to intersecting negative social relationships which combine to increase overall vulnerability. These included relationships with their partner, with the police, with other WWUD, with persons in prisons, with their family, with their children, and with their healthcare team. The interviewed women described multiple forms of stigma and suffering which they had not forgotten. Guilt about their parents and their children was the most striking characteristic of the way in which women shared their life narratives. This moral burden was a specific element of gender. Interviewed WWUD believed that they had to “hide” and to be “guilty”, two features creating the basis for very strong self-stigma. By deviating from social and moral norms, WWUD had to face social sanctions (e.g., rejection by their family, exclusion from socializing in their neighbourhood, disqualification from motherhood) and criminal penalties (double punishment for substance use and for sex work). Only a few WWUD in our study were less exposed to these vulnerable situations: those who only occasionally consumed drugs and those with a healthy financial situation.

The interviewed PWUD (i.e. women and men) highlighted gender-based differences in terms of personal and contextual barriers to accessing HR services. First of all, women were afraid of stigmatization. Added to this were the burden and constraints of parenthood, and the fear of being ‘outed’ as a PWUD. In turn, these elements constituted barriers to HR service utilisation and access. These barriers were more experienced by women than by men. In addition, women had high expectations in terms of the range of psychosocial support: they called for psychological support, empowerment-based activities, material assistance, etc. The study interviews highlight that the reasons why men did not seek care were different from those of WWUD. Men were afraid that methadone would be ineffective, that it would have side effects on their libido, and that they would relapse. They also stated that they would not have enough time to come to CEPIAD because of work-related activities.

These gender-related differences in utilisation of and access to HR services are illustrated in the following comments:

*I was told about CEPIAD*,* but I didn’t go there; I don’t feel comfortable going there. I don’t want my child to find out tomorrow that I was using [drugs] (…). I don’t want to go there for fear of meeting people who know me. (…) (Coura*,* 34 years old) WWUD*.

*I saw a guy on methadone and he’s married. Do you know what he told me? (…) He told me that methadone leads to sexual impotence. He even had to stop taking it recently. I’m willing to go to CEPIAD*,* but not to take methadone. If I decide to stop [taking drugs]*,* it’ll be to never again take any product. In my opinion*,* methadone is a drug too*,* and what’s more*,* it has a stronger effect than a [heroin] fix. (Mactar*,* 43 years old) MUD*.

Women generally expressed needs that were somewhat different from those of men. They called for outreach care, as they could access it more easily. Furthermore, outreach would take account of their family-related constraints. They also wanted care that would be more sensitive to their bodily and sexual and reproductive health needs. Moreover, interviewed WWUD who attended CEPIAD and who were aware of its services requested social activities dedicated to women (income-generating activities, support for improving self-esteem, self-support through peer groups, support for caring for their children). They also underlined the importance of confidentiality.

Irrespective of their social and drug consumption profiles, the relationships the interviewed WWUD had with HR services were far from being similar. We shall examine this issue in the following paragraphs.

#### An absence of consideration of the diverse profiles of WWUD

CEPIAD’s care offer is based on a specific treatment protocol. Other care and prevention services can be provided on-site (i.e., at CEPIAD’s premises) in addition to the standard HR offer. In terms of WWUD, CEPIAD’s outreach teams intervene directly in drug consumption spaces to reach this population, to provide them with information and prevention tools, and to direct them to the centre. However, our field data show that WWUD in Senegal have diverse socio-demographic profiles and that these profiles are linked to the consumption of different products (crack, cocaine, heroin, diverted medications, tramadol, cannabis and alcohol). Moreover, the consumption patterns and contexts for these substances differ. The difficulties of CEPIAD’s current HR offer in reaching WWUD partly stem from a lack of consideration of their diversity and their specific psychosocial vulnerabilities.

From the data collected during interviews with WWUD and from observational data, we distinguished four profiles:

- Profile (1) Young women from the nightlife and entertainment world. These WWUD consumed alcohol and cocaine in a party context. Most were economically independent (i.e., not living in social precarity) and stated they are able to manage their drug consumption. They believed that drug use did not influence their state of health.
- Profile (2) Young women from working-class neighbourhoods and sex-worker (SW) associations. They were mostly cocaine/crack users. Some were intermediaries between dealers and users on the drug scene.
- Profile (3) Women known in the PWUD community as “former junkies”. They had used heroin for several years and most were on methadone treatment. Some had an intersectional experience, being also sex workers and living with HIV/AIDS.
- Profile (4) Women dependent on medications and isolated. Some of these women were receiving methadone treatment at CEPIAD but overused tramadol, a painkiller prescribed to relieve severe pain linked to conditions such as sickle cell anaemia and scoliosis. These women did not belong to any marginalized social category.

Women from profiles 1 and 2 were for the most part cocaine/crack users. They did not frequent CEPIAD and did not have access to any decentralized HR service, which partly explains the low number of WWUD in CEPIAD. They often had intersectional backgrounds (i.e., sex work, HIV/AIDS), and needed access to safe HR spaces and services where confidentiality was prioritised, as well as outreach-based sexual and reproductive health services.^4^

Women with profile 3 generally appreciated the care provided at CEPIAD. They welcomed the therapeutic effects of methadone which allowed them to escape the constraints and everyday risks linked to heroin addiction. However, they considered the care services offered to be insufficient, particularly women-specific activities, dental and gynaecological care, socialization activities. In addition, their journey to better health through methadone treatment brought other expectations that no health or social service could currently meet, such as support for professional reintegration.

The characteristics of social marginalization for women from profiles 1 and 4 were different from those of the majority of the WWUD interviewed. For these two profiles, stigma was the main obstacle to seeking care. They rarely expressed expectations in terms of social support. Instead, they had a greater need for prevention interventions against addiction and overdoses in a context where the availability of naloxone is still limited in Senegal. Women in profile 4 report needing psychosocial support and specific self-support to discuss issues surrounding addiction to pharmaceuticals, which was usually the consequence of receiving prescribed drugs.

These four different profiles show the need to provide tailored HR responses and to go beyond traditional stereotypes of WWUD as “mothers” and “prostitutes”. The intersectionality of these women’s profiles must be taken into account to overcome barriers related to stigmatization, discrimination and social exclusion, and to ensure equitable access for them to health and support services. Profiles 1 and 2 require more outreach activities and need specific spaces dedicated to women, either to avoid their invisibilization in terms of the current HR offer, or in contrast, their exposure to criticism in mixed-gender environments.

#### HR stakeholders’ focus on infectious vulnerability to the detriment of social vulnerability

The interviews conducted with stakeholders who implement HR highlight that they perceived women to be more vulnerable than men, when taking all the following social conditions into account: modes of injecting drug (i.e., injection itself and the sharing of injecting equipment), sex work, social stigma and criminal sanctions, and finally, social precarity. In addition, they face harsher repression than men and are exposed to societal expectations of submission that make them vulnerable to exploitation. Global health institutional HR policies and programmes are mainly focused on prevention and care for addiction and infectious risk. When applying them, institutional actors primarily target women who inject drugs (WID) instead of WWUD more generally. However, drug injection does not reflect the reality of collective needs on the ground in Senegal where there are relatively few WID. Although care providers are aware of this, the focus on injecting drug use is justified by HIV prevention issues. This is highlighted in the following extract from an interview with a member of the Senegal Interministerial Council for the Fight Against Drugs (CILD):

*Women are generally more affected by HIV (…). ‘WID’ is an internationally recognised term. Indeed*, *it’s injection that causes these problems of blood-borne diseases*, *with the sharing of syringes and other stuff…. and that’s why we must focus on contamination. CILD member*.

IDUs constitute one of the “key populations”^5^ for the control of the HIV, HBV and HCV epidemics in Senegal. However, due to the low rate of injecting practices in the country, and the difficulty to define strict categories that reflect actual drug use behaviours, the term ‘IDU’ is used to cover users of all drugs that can be injected, irrespective of the actual route of administration used in the drug scene. For example, the term IDU in Senegal also covers PWUD who smoke heroin. WWUD are often doubly concerned by this categorization, as they have an intersectional IDU-SW profile (irrespective of whether they are simply considered to be SW a priori, or whether they do in fact work as SW).

Furthermore, our empirical data show that irrespective of the type of drug consumed (e.g., alcohol, injectable drugs) and the mode of consumption (e.g., injecting, smoking, eating, inhaling, etc.), sexual interactions with PWUD (whether romantic or related to prostitution) can be considered as carrying varying degrees of HIV infection vulnerability. For example, most WWUD in Senegal use cocaine (or crack), which is not injected but smoked. Furthermore, some consume alcohol in the world of nightlife and entertainment, and are thus exposed to risky practices (e.g., unprotected sexual intercourse under the influence of substances, prostitution). Accordingly, sexual relations expose women more often to the risk of HIV acquisition than drug injection.

HR interventions in Senegal include the provision of condoms, as well as needle exchange and opioid substitution therapy programmes to limit the transmission of infectious comorbidities. However, some experts believe that HR interventions must also embrace other issues, given that all actions aimed at improving the quality of life of PWUD are relevant [18]. Indeed, this is exactly what HR stakeholder observations and discourses communicated during our survey.

Community stakeholders in our survey also perceived that WWUD were more vulnerable than men who use drugs. During HR interventions with PWUD communities, they observed that WWUD were more likely to live in social precarity, that they were less accessible to outreach teams, and that they were more likely to be HIV positive. They criticized the use of the term IDU in their field of work - which they felt abusive - as it does not adequately reflect the wide diversity of WWUD profiles. The use of terms like ‘IDU’ was perceived as restrictive, as it limits the analysis to HIV/AIDS-related issues, while similar challenges may affect women who consume other substances, such as alcohol. It also highlights the importance of considering broader themes related to drug use among women, including the impact of certain substances on sexual behaviors and their relevance to HIV risk. The statements collected suggest that HIV prevention is the initially necessary and relevant “gateway” to advocate a drug policy that is based on health and to bring HR policies to the local level. The respondents also discussed the need to move towards a more global approach to the various issues affecting drug users. They advised to take into account the diversity of user profiles, particularly women, who constitute minority populations in structures providing support to PWUD. Focusing only on their vulnerability to infection is not always sufficient to reduce their global social vulnerability, because, as Desclaux et al. (2011) put it: “the ‘health rationale’ is not enough to change social relationships if relationships of power and cultural logics persist” [19].

Stakeholders from CEPIAD and from NGOs in Senegal who work with WWUD understand that the multidimensional nature of this population’s needs goes beyond their vulnerability to HIV infection. However, the services these stakeholders can provide are very limited and have little impact on social vulnerability. They are also constrained in their work by the conceptual tools used in the field of intervention, such as the notion of ‘IDU’ (see previous paragraphs) which focuses on the fight against HIV, thereby limiting the support that can be provided to PWUD who have intersectional vulnerabilities, such as women.

### The gender approach in the Senegalese healthcare system: achievements and limitations

#### A system under construction, coherent but fragile

In Senegal, health-based (e.g., doctors, addiction specialists), community, and institutional (e.g., ministries, NGOs, etc.) stakeholders are all involved in creating gender-specific HR initiatives.

With regard to health-based stakeholders, CEPIAD and its HR programme were established to respond in particular to the needs of persons with an accumulation of different medical vulnerabilities (i.e., with several pathologies) and social vulnerabilities (i.e., dependents, poor). CEPIAD promotes “integrated care”, an innovative and original strategy which recognizes the impact of social, economic and environmental factors, as well as gender inequalities, on PWUDs’ lives. In this way, integrated care differs from other strategies that only provide support for addiction. CEPIAD’s HR service offers somatic and psychiatric specialist care for addiction, opioid substitution treatment with methadone, counselling, screening and treatment of comorbidities (HIV, hepatitis), empowerment activities, and prevention measures (e.g., needle exchange programme and condom distribution). This service is decentralized in four regions: Thiès, Diourbel, Kaolack and Ziguinchor. The gender-specific HR programmes offered by CEPIAD are supported by the National AIDS Council in Senegal (CNLS), the French government agency for research on HIV/AIDS, viral hepatitis and emerging infections (ANRS MIE), SOLTHIS NGO 6 through the ATLAS Project^7^, and the United Nations HIV/AIDS programme (UNAIDS).

With regard to community stakeholders, the NGO ‘Enda Santé’ (supported by the Open Society Initiative for West Africa OSIWA) and the National Alliance of Communities for Health (ANCS) are the two main stakeholders implementing gender-specific HR initiatives in Senegal. These two structures have strong community roots on issues related to key populations. The interviews we conducted with stakeholders from both structures highlight that the gender factor takes a central place in the overall development of their strategies and interventions for key populations. Community support is oriented towards capacity building of PWUD, including WWUD. ANCS’s objective is to strengthen health systems through community empowerment. Interventions for PWUD in general have been made possible thanks to support from the Global Fund to Fight AIDS, Tuberculosis and Malaria, the Frontline Aids partnership, and the London School of Hygiene and Tropical Medicine. Enda Santé supervised a project to set up income-generating activities (IGA) for PWUD associations, and the first WWUD association in Dakar, called “Femmes engagées” (Committed Women), which was financed by OSIWA.

At the institutional level, the CILD - which was created in 1997 - is responsible for organizing the fight against drug addiction and trafficking. It integrates a gender-sensitive approach into its directives and activities. The CILD reports to the Ministry of Home Affairs. It comprises 18 ministries; six civil society organizations are also members.

HR health stakeholders’ (i.e., medical professionals, paramedical professionals, and social workers) and NGOs’ discourses in relation to WWUD highlighted the difficulty in reaching women in the interventions they currently offer, particularly since the ‘night world’ has been moving from nightclubs and bars to private homes and meetings organized over the internet. Some stakeholders stated that once they have connected with WWUD, women request economic support to meet the travel costs to CEPIAD, and interpreted this request as a lack of willingness by WWUD to come for care. Some also blamed the lack of leadership among women in the PWUD groups they support, with no one wanting to take on the role of leader. Whether in terms of healthcare or community projects, stakeholders agreed that they also find it difficult to mobilise WWUD for activities which specifically target them. The following excerpt from an interview with an NGO stakeholder discussed the issue of the invisibilisation of WWUD and the difficulty in mobilising them:

*I believe that one of the main problems that we’ve had is mobilization; it is the inability of women to take responsibility for themselves as consumers and to work alongside their peers. I believe that for some time now*, *things have started to evolve timidly (…). NGO project manager*, *interviewed in 2020*.

This difficulty in reaching WWUD was also reported by stakeholders who provide care. To increase the participation of WWUD in healthcare provision, CEPIAD’s team had tried to set up activities to attract them. However, they are constantly faced with the difficulty of reaching and mobilizing this population, as one addiction specialist testified:

*We still have very few gender-specific activities… each time we initiate something - like the sewing workshop that we had planned – it’s the men who take over*, *because no women come. Social opinion contributes too*, *a little*, *to stigmatization. Addiction specialist*.

The stakeholders interviewed considered that the difficulty in reaching WWUD was linked not only to stigma and self-stigma but also to gender relationships which reshape mixed activities to the disadvantage of WWUD. They also blamed the absence of programmes which WWUD might find interesting. The fact that men took over initiatives aimed at women indicates that even when opportunities are created for women, they often let men take control. With respect to the sewing workshop cited, sewing is an activity that requires specific skills. In Senegal, it is a work sector dominated by men. However, the organization of women’s groups and associations is increasingly enabling women to get involved in activities previously monopolized by men, such as sewing and embroidery. The development of awareness strategies on empowerment capabilities could encourage the participation of women in sewing workshops at CEPIAD.

NGOs refer WWUD to CEPIAD for care, mostly through screening. The criticisms levelled at CEPIAD by the WWUD interviewed in our study regarded the limited capacity of the centre’s care and support offer to ensure sustainable funding for the women-specific activities proposed, and the absence of financial support for the treatment of diagnosed pathologies. This issue is linked to the capacity of specific systems and services to address the overall health needs of people, a structural problem that has been widely explored among people living with HIV. Indeed, while ART is free of charge in Senegal, treatments for other pathologies represent substantial out-of-pocket costs to patients. This reality casts doubt on the usefulness of screening for these pathologies in a context where people cannot pay for treatment after diagnosis. Moreover, these costs are the result of the “vertical” organization of services and the availability of international funding for a given population, a problem that can only be resolved by another method of health financing, such as Universal Health Coverage.

Furthermore, since 2016, in an attempt to combat the invisibilisation of women in HR programmes, and despite the difficulties encountered, various stakeholders providing HR services in Senegal have tried to implement gender-sensitive initiatives, which we list in Table 1.

**Table 1:** Interventions specific to women who use drugs in Senegal

Institutions providing support to PWUD	Intervention area	Objectives and targets of gender-based activities	Activities implemented	Duration of intervention		Implementation	
			Activities	Start	Duration		
						Facilities	Difficulties encountered
CILD	Defines national policy to combat the abuse and illicit trafficking of drugs. Coordinates the actions of the various State services in Senegal in, among other things, the fight against drug addiction and illicit drug trafficking	To take the gender factor into account in the fight against drugs	Women’s Day (national drug awareness week)	2019	1 day annually	Collaboration with stakeholders in the HIV response	COVID-19 pandemic. Technical terms are difficult to translate into Wolof. No access to WWUD to involve them in the organization of the activity
CEPIAD	Offers integrated treatment for addictions (HR, Opioid Substitution Treatment, Management of comorbidities, Social activities)	To increase the attendance of women at CEPIAD To promote access and maintenance of care for women	Gynaecological consultations Gynaecological follow-up Women’s support groups Zero discrimination day for women who inject drugs	2017 2016–2019 2019–2021 2020	3 months 3 years 2 years 1 week	Financial support from donors Support from Outreach peer workers for the mobilization of women	Sustaining activities at the end of projects Mobilizing and reaching women
ANCS	Encourages, supports and strengthens community participation in public health activities for populations in general and in particular against HIV/AIDS.	Strengthening health systems through community empowerment (including women)	Establishment of a self-support group for drug users in Mbour. Strengthening the capacities of communities through the involvement of peer workers in projects (the PARECO Project)	2017 2017–2020	3 years 3 years	Long experience with communities	Bringing women together. The transportation costs for women. Lack of female leadership. Funding.
ENDA Santé	Supports populations - particularly vulnerable groups - in defending their rights to access to information and adequate health services.		Support for the establishment of a the first WWUD association in Senegal “Engaged women”	2021	18 months	Financial support from OSIWA. Capitalizing on experience with other PWUD associations	Female leaders had expectations beyond the capacity building dimension proposed by the project to support the creation of the association (e.g., leaders expected remuneration/a salary)

Looking at the objectives and approaches of these different interventions, we can distinguish two categories: (1) those offering care targeted specifically at women, based on their specific needs (i.e., in comparison to men); and (2) those based on capacity building for women, prioritizing their autonomy.

#### A care approach that specifically targets women

The inclusion of gender issues in political and strategic documents demonstrates a desire by HR stakeholders to take into account the vulnerabilities of WWUD and to reduce inequalities compared to men. However, the notion of intersectionality, which combines gender vulnerability and social vulnerability, is not at the forefront of this approach.

CEPIAD and CILD provide an intersectional approach to WWUD-specific support. Taking gender into account is a key directive of their strategic plans. Gender issues are included in all CEPIAD and CILD projects and programmes. The impact of this approach can be seen in the daily functioning of CEPIAD, for example through the offer of gynaecological consultations and women’s focus groups.

A member of the CILD team explained that the gender dimension is important for them. When the CILD team organizes training workshops with the forces of order, women are represented. They also organized a training workshop on drugs exclusively for the Badjenu Ngokh^8^ because CILD’s strategic plan includes a programme with them, given their important role in the society. Approximately thirty women participated in that workshop. The CILD also organizes a ‘Women and Drugs Day’ during the yearly drugs awareness week:

*I’ll give you the example of the airport anti-trafficking unit which comprises members of the police*, *customs officers… This unit does not include women. We plan to change this*, *to ensure that women are represented*, *especially for body searches. For women transporting drugs*, *it is not normal for men to search them. (…) And it’s the same thing too for the mixed-gender container control unit which does the same work at the port level*, *so that the gender dimension is taken into account at that level too. Now*, *that’s a project!*

Since it started its activities in 2015, the small proportion of women on the CEPIAD patient list has always challenged the centre’s various teams to implement activities that women find interesting and useful. This is why one of the directives of CEPIAD’s strategic plan is “*the promotion of an ethical*,* socioeconomic and political legal environment which favours the respect for human rights and gender in the implementation of the set of specific services for women who inject drugs”*. In addition, most WWUD on methadone treatment at CEPIAD (i.e., approximately 10 women in 2022) are involved in the centre’s outreach field team, with a view to reaching a larger number of women who do not yet attend the centre. This strategy is seen as positive discrimination against women at CEPIAD.

##### Gynaecological consultations

In Senegal, HR programmes primarily focus on distributing prevention materials, such as condoms and injection tools, as well as screening and treatment for sexually transmitted infections (STIs). However, they rarely integrate other essential dimensions of sexual and reproductive health (SRH). Recognizing the specific needs of women led CEPIAD to start providing an SRH service that includes gynaecological follow-up, screening for STIs, and screening for cervical cancer. Initially, gynaecological consultations were set up through the CODISEN project and the CEPIAD/Enda Santé partnership established in 2018. Using a mobile clinic to reach WWUD, these consultations were very successful in terms of the number of participants. They also helped CEPIAD connect with WWUD to attract them towards its HR services, beyond providing them gynaecological care. However, because of a lack of funding, the consultations could not be provided for more than a year, CEPIAD could not offer follow-up after its end to women who had received gynaecological care during the CODISEN project. The limitations met were mostly linked to resources:

*The constraints were the sustainability of activities*, *and the management of [para-clinical] tests because many [women] had gynaecological complications*, *and it was necessary to go further and do ultrasounds*, *smears*, *and even [surgical] interventions. And that was the main constraint… Addiction specialist*.

This extract reveals the difficulties encountered by CEPIAD in continuing to offer SRH services to women despite being very much appreciated by the women interviewed. Moreover, it reflects the problem of the sustainability of health interventions when funding is occasional and comes from global health organizations or bilateral cooperation. The lack of financial leadership at the national level hinders the development and continuity of effective, long-term interventions.

##### Women’s focus groups

At CEPIAD, complementing individual support with collective support is part of the centre’s approach to health promotion. In 2017, women-only focus groups were organized every fortnight with the psychologist at CEPIAD so that they could discuss the vulnerabilities that affected them. The idea was to motivate women to attend the CEPIAD by choosing relevant topics for discussion. However, the initiative did not last. Moreover, as part of the ATLAS project, focus groups for women were initiated by the centre in collaboration with the NGO SOLTHIS, as highlighted by these comments from a CEPIAD addiction specialist:

*With ATLAS*, *we started in 2019*, *it [the project] was for 3 years. ATLAS was generally about doing things that would complement what we do for the Global Fund… so the idea was to fill the gap we have with women*, *and that’s why we proposed women-only focus groups. We also suggested [doing outreach] nights out in order to meet women [i.e.*, *WWUD] who frequented bars*, *etc… The topics of discussion mainly revolved around HIV. Then we delivered self-tests*, *and while we were doing that*, *we took the opportunity to raise women’s awareness about the risks associated to their consumption patterns (…) It is really a gateway to care and to maintaining them in care. Addiction specialist*.

The themes developed in the focus groups at CEPIAD essentially concern drug consumption in connection with infectious pathologies. Themes are rarely chosen by the women themselves. Beyond raising awareness, the focus groups lead women to consider CEPIAD as a new space for support and for socialising with people who share common problems. There, women can establish relationships that continue outside the centre.

##### Annual women and drugs day

Each year, the CILD organizes a national drug awareness and mobilization week. Like for the international day which celebrates the fight against drugs, Senegal has opted for a week punctuated by several activities including panel discussions, conferences, awareness raising events, and hikes. In the 2019 edition, a day was dedicated to women. Discussions that day regarded the legal framework of drugs and drug use, addiction, and the modes of HIV/AIDS transmission among WWUD. CILD decided to integrate this Women and Drugs Day into the national drugs awareness week to raise greater awareness of the issue of gender in terms of drugs. The respondents indicated that while awareness messages are mainly broadcast in French - the country’s official language (widely used in the fields of education, administration and the media) people who have limited French language skills and who speak Wolof, Peul, Serer, etc., may find it difficult to understand these messages.

Besides the language barrier, the absence of any representation of a WWUD association in official committees such as CILD was also seen as a reason for the lack of participation of women. However, a CILD team member stated that the recent creation of a WWUD association [referring to ‘Femmes Engagées’] provided an opportunity to start a collaboration.

In December 2020, CEPIAD, in collaboration with UNAIDS, organized a “Zero discrimination week dedicated to women who inject drugs”. This activity was part of the general framework of the Zero Discrimination Day^9^ organized each year by UNAIDS to raise awareness of the need to act to eradicate gender inequalities. Due to the COVID-19 pandemic, this activity was postponed from the the annual Women’s rights week in March to the International AIDS Day in December, and extended to last one week. The CEPIAD team defined the content of the activity with WWUD to meet their needs:

*We asked them and they thought that learning how to make soap might be useful for them*,* and so we set up soap making workshops to introduce [the process to] them so that they could sell soap and make money. Apparently they really liked it; they were happy. Addiction specialist*.

**Fig. 1:**
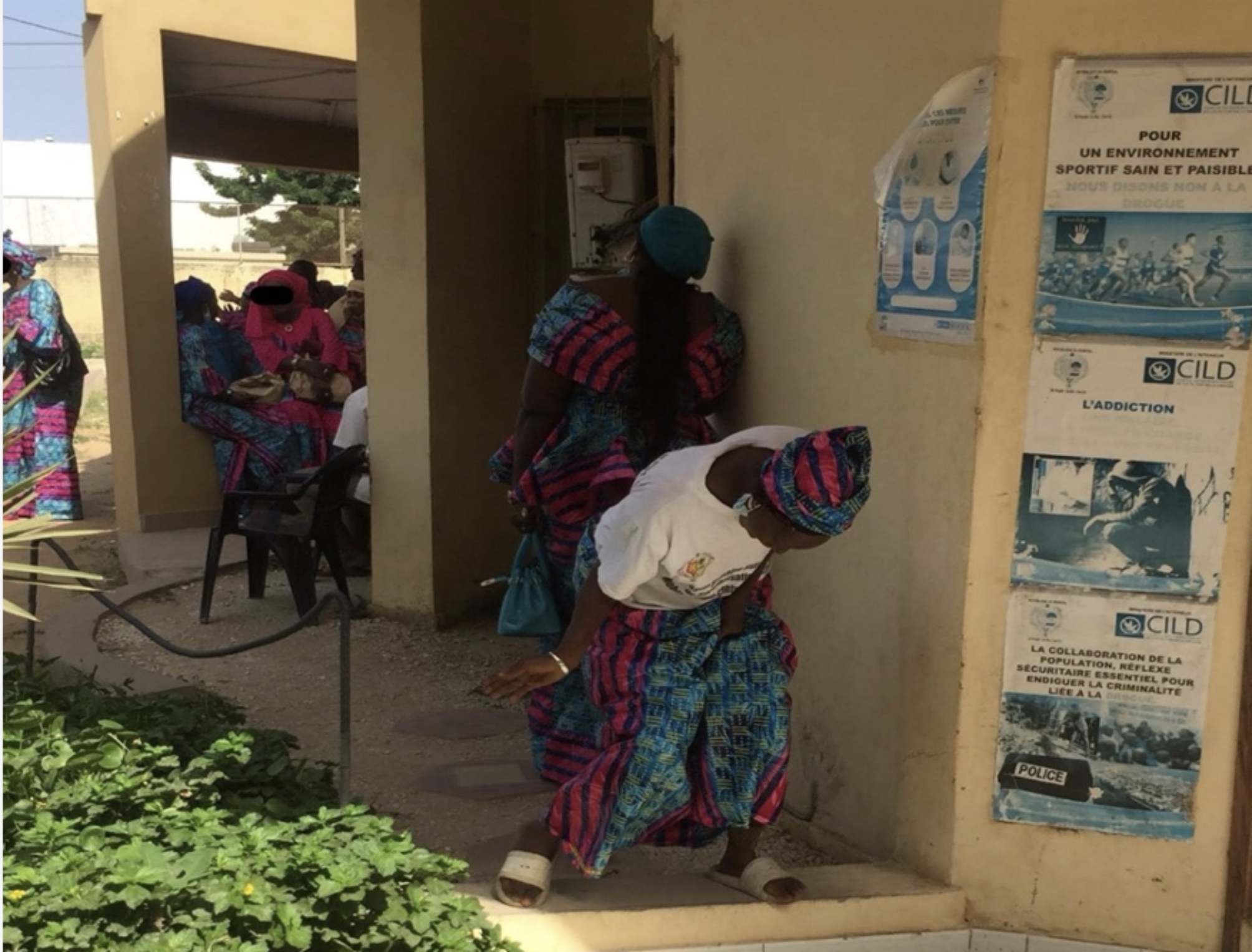
Women wearing nirolé (garment made from a material of the same name) during the Zero Discrimination Day 2020 campaign at CEPIAD (Dakar)

During the 2020 Zero discrimination day, women were able to access HIV and hepatitis C screening services, have a gynaecological consultation, including cervical cancer screening, and obtain information on addiction. They also learned how to make soap. When organising this day, the stakeholders considered gender in an intersectional way, taking into account the specific social precarity of women. This Zero discrimination day, the first of its kind at CEPIAD, was appreciated by the women interviewed because it was planned in collaboration with them. At the closing ceremony, they were dressed identically in “nirolé” (name of a specific type of dress in Wolof), magnificent, and happy to present the products they had made themselves (Fig. 1). In her address, the women’s representative highlighted that they appreciated this initiative and wished it to become a sustainable activity, as women would be able to obtain information and improve their capacity building.

#### An approach based on strengthening agency

The NGOs Enda Santé and the National Alliance of Communities for Health (ANCS) implement gender-sensitive initiatives, with strong community roots on key population issues in Senegal. Their strategies, which are supported by various partners, focus on community empowerment and capacity building of PWUD, in particular WWUD, with projects such as the creation of associations and income-generating activities. In the two subsections below, we discuss peer-to-peer support and the emergence of women in the world of community associations.

##### Peer-to-peer support

The ANCS has more than 10 years of experience supporting drug user groups, as a member of its team indicated:

*Around 2005*, *the ANCS had already set up a self-support group called APSUD*^9^*and there are still survivors of this group who are still here; and that’s important in the history of the ANCS*, *because there was [only] a smattering of support before the arrival of CEPIAD. But we had to suspend [it] due to lack of funding. Project manager*

ANCS’s objective is to organize communities to raise issues that affect them. This is why its strategy consists of involving peers in programmes and organizing them into self-support groups.

A large component of the Global Fund covering 2016 until 2024 (Grants NMF 1 and NMF 3) is reserved for PWUD. The Jacques Chirac centre, a drug information and awareness structure located in the suburbs of Dakar, is responsible for implementing Global Fund-related activities for PWUD in Senegal. Specifically, it provides legal services, services for socialising, and psychosocial services, as well as community prevention services to PWUD. In June 2024, the stakeholders who implement these activities met together to reflect on how to implement gender-sensitive initiatives into their annual programme. The Global Fund has also made it possible to provide individual support in response to requests from women (peer-mediator salaries, housing assistance, financial support for purchasing medicines and for health check-ups).

From 2017 to 2019, the ANCS implemented the Regional Harm Reduction Programme for People who inject drugs (PARECO). Financed by the Global Fund, this project was implemented in five West African countries (Burkina Faso, Cabo Verde, Ivory Coast, Guinea Bissau and Senegal). Within the framework of PARECO, bio-behavioural studies on IDU were conducted, and innovative HR models were implemented with PWUD as peer workers. WWUD were prioritized during recruitment and several WWUD worked as peer-mediators in the project.

From 2015 to 2020, in collaboration with Frontlines AIDS, the ANCS ran a specific community-based HR programme for PWUD called BUZA, with the objective of creating officially recognised, authorised PWUD associations. A partnership was started with the London School of Hygiene and Tropical Medicine to initiate participatory research with peer workers [20]. The programme aimed to identify PWIDs, to mobilize them, to support them in setting up self-support groups, and to supervise them so that they could organize themselves in a way that was officially recognized. Thanks to BUZA, 10 officially recognized associations have already been created, and 10 others are still waiting official recognition. In the latter group, only one self-support WWUD group has been set up (in the Petite Côte region located around the cities of Saly and Mbour).

The ANCS has also worked to mobilize women in Dakar, Thiès and Saint-Louis. Its approach in this regard consists of setting up peer support groups, improving capacity building in association management and involving PWUD peers (women and men) as peer workers in projects. As part of this approach, the PWUD mobilized in ANCS programmes gradually position themselves as community leaders. Self-help groups consist of people suffering from similar conditions or circumstances being brought together so that they can defend their own interests, receive support, and help each other through the sharing of knowledge, experiences, and coping strategies, with a view to overcoming drug-related problems.

*They [WWUD] have been invited several times to reflect with us*, *to express their needs*, *and the ANCS has endeavoured to take care of these needs (…) Each time someone among them expresses a need in writing*, *the ANCS tries to take care of it. If you go to Mboro and Saint-Louis*, *we don’t yet have groups with a critical mass of women*, *but we encourage men to empower women; so*, *we’re still at the early stages but we hope that it will evolve (…) Project manager*.

This is why it was not uncommon that women interviewed in our study asserted their involvement in WWUD-targeted activities in terms of the slogan: *“Nothing about us without us”*. The interview extract below shows that this strategy is important for empowering communities, but that once these groups are formalized, they have critical needs in terms of financial support and support to implement activities.

*We know that there is still a lot to do because*, *you know*, *in these programmes*, *we have taken people out of difficulty but we have [also] created other difficulties. When someone was in their ghetto and didn’t necessarily need to be well dressed uh… now that we’ve made them into someone who frequents ‘classy places’*, *[new] needs emerge. When you create a leader*, *you don’t have a lot to give to them. That’s a problem. So the lack of funding poses a problem but so too does the lack of any significant motivation [subtext: monetary reward] for the leader. Project manager*.

Accordingly, establishing relationships with women by responding to their social vulnerability creates new difficulties linked to the limitations arising between support for autonomy and assistance based on a paternalistic or charitable approach. In a context where material resources are limited, this question raises a major issue in the fight against social inequalities.

##### The emergence of women in the world of associations

As mentioned above, among the 20 PWUD associations which have been established to date with the support of the ANCS, 10 are now officially recognized. The latter are mainly supported by Enda Santé to develop HR activities (awareness) and Income Generating Activities (IGA) with funding from OSIWA. Since 2018, three of these associations (two in Dakar and one in Mbour) have received a grant to initiate activities as part of the *Geum sa bopp*^10^ project: Santé Espoir Vie (SEV), The Association for the Fight against Drug addiction, TB, HIV/AIDS and stigma (ALT 2 S), and finally, Sauver Ma Santé (SMS). Although all three have women among their members, most members are men. Furthermore, few women have positions of responsibility.

In this context, a number of women advocated and demanded support for the establishment of a women’s association during a consultation meeting of various stakeholders at the OSIWA headquarters in Dakar. They condemned the fact that women had little power and little say in existing associations, where they were rarely members of the board of directors. They explained that this prevented them from expressing and carrying out their social and political demands. Following this advocacy, OSIWA asked them to come up with a project for women and to apply for funding. Enda Santé played a supporting role but it was the women themselves who took on the responsibility of conducting this initiative to represent WWUD in Senegal:

*In reality*, *it was the women who wanted to form an association; they went to get their own [official filing] receipt [for the creation of the association] and all that. Our approach now is that we don’t impose anything on them*, *we supervise them by trying to get them to ask themselves the right questions*, *to make the right decisions…etc. That’s it*, *we are really talking about support but not substitution. (…) Ideas come from them*, *their development must also come from them. Project Manager*.

The project presented was to support the establishment of a WWUD association, and to build members’ capacities in terms of managing an association as well as strategic development to look for financing and partnership. The project, which was initially focused on material aid, includes several activities, including a study on the vulnerability of WWUD in the Petite Côte, consultations to assess training needs and members’ expectations of the association, personalized support for leaders according to their training needs, community meals twice a month organized by the women themselves, monthly meetings between Enda Santé and the women, exchange workshops, meetings with leaders of other key population associations to understand their working models, etc. The association is called “Femmes engagées” (Committed Women); it brings together WID (and former IDU on OST or not). The founders decided themselves to limit members to the IDU category at the start of the association before integrating other WWUD profiles, as the president of the association explained:

*Because we are at the beginning*, *we are experienced in terms of consumption. (…) If the need arises for other consumers of any drug whatsoever*, *be it alcohol or cannabis*, *we will do it. (…) We will then see how to add the others. We already have a lot of work to do with women who are IDU” Head of the Femmes Engagées association.*

Members of the association believe they would have the necessary skills to expand their membership, if they were provided support to do so. The association ‘Femmes engagées’ was officially recognised in 2020 and capacity building activities with Enda Santé began in February 2021 for a period of 18 months.

With the advent of the ‘Femmes engagées’ association, WWUD in Senegal are beginning to mobilize to overcome their invisibilisation, help each other, and empower themselves. This process is based on the engagement of a group of WWUD leaders who want their specific needs to be taken into account as PWUD, as women, and in their specific social context.

## Discussion

Our study shows that WWUD in Senegal experience discrimination and stigmatisation associated with gender, addiction (the double stigma of being a PWUD and a WWUD), structural conditions, and biopsychological vulnerabilities, and that all these factors limit their access to and retention in care. It also shows that that these factors are intersectional. Accordingly, any intervention focused on improving access and retention must take into account intersectionality. We found that women feared stigmatisation more than men. The gender factor was particularly present in terms of stigmatisation linked to gender norms. Moreover, family exclusion because of their drug use deprived WWUD of opportunities for social interaction. This process of exclusion negatively impacted WWUD’s relationship to care, and they tended to distance themselves from care structures. At the INHSU 2023 conference, a presentation on women and drugs in Côte d’Ivoire found similar findings to ours. Specifically, the authors indicated that WWUD in Abidjan experienced social and health vulnerabilities, and faced barriers such as a repressive political environment and stigmatisation, which hindered their access to health services [21]. With regard to structural and systemic barriers, a study conducted in Kenya revealed that long distances, lack of confidentiality, service fees, multiple appointments, poor communication with healthcare providers, and the providers’ lack of understanding of women’s needs are key factors hindering women’s access to health services. Community-based services, including local drop-in centres and reception facilities, help mitigate these barriers by building trust, informing women about their health and rights, connecting them to harm reduction (HR) facilities, raising healthcare providers’ awareness of the needs of women who inject drugs, and integrating sexual and reproductive health (SRH) services for women into community HR activities [22].

Our study found that some gender-sensitive interventions already exist in Senegal, mainly implemented by CEPIAD, CILD, ANCS and Enda Santé. These stakeholders recognize the different needs of women and men, as well as gender power dynamics. They understand and take into account the fact that gender influences experiences which in turn shape women’s use of HR services. Their interventions focus on women and empowerment. Could the services they provide be more *gender responsive*^11^?

In Senegal, access to care and maintaining care is often more difficult for women than for men, particularly due to greater stigmatization and insufficient consideration of their needs. These two elements reflect the socioeconomic gender inequalities in Senegalese society. A study on the involvement of Senegalese association-based stakeholders in HIV research showed that before being able to participate in research studies, these stakeholders had to first resolve several material difficulties (e.g., financial aid) [23]. The severity of these difficulties would in turn affect the magnitude of the obstacles they create to community involvement in a given context.

HR stakeholders in Senegal are slowly beginning to take these factors (i.e., access to care, greater stigmatization, etc.) into account when developing interventions and WWUD-specific activities are gradually being offered. However, women are not included right from the beginning of the activity reflection process; doing so would make it possible to become more aware of their specific needs. Nonetheless, the HR efforts made to date by stakeholders show incremental progress. It is important to point out that this gender-based advocacy for WWUD has no support at the international level in Africa, something which is perhaps due to the fact that messages from African Francophone countries are often not heard in Anglophone circles. The Civil Society Forum on Drugs, created at the end of 2023 by the African Union (AU), needs to insert gender-specific aspects in HR programs as part of their agenda.

With regard to current interventions in Senegal, which are mainly focused on IDU, it is important to move from a male-centred model to an approach which also considers female needs, then to an approach which considers relationships between men and women, in the context of gender relationships. While IDU men in our study expressed needs related to their negative perception of the effects of methadone treatment on sexuality, no sexual health initiatives in Senegal currently includes this population or addresses couples. The creation of drug use awareness-raising programmes including the partners of PWUD, and the development of online resources dedicated to couples where at least one person is a PWUD, could help clarify the links between sexuality and drugs.

The objectives of HR in Senegal are to reach a greater number of WWUD, to encourage them to seek and stay in care, and most of all, to support them towards self-empowerment. Current women-specific interventions are inspired by internationally-recognized HR models (gynaecological follow-up, days dedicated to women, support for PWUD associations). However, they are partly gender blind and HIV-focused^12^. In other words, current interventions are confined to women who are IDU, and the issues tackled mostly concern vulnerability to HIV. This means that users of non-injectable substances (alcohol, cannabis, etc.) are excluded, and that gender diversity and the intersectionality of WWUD profiles are not taken into account.

We identified four WWUD profiles: (1) young women from the nightlife and entertainment world (the circulation of money milieu), (2) young women from working-class neighbourhoods and from sex worker associations, (3) Women known in the PWUD community as “former junkies”., (4) isolated women dependent on pain medication. These different profiles should be taken into account by diversifying HR approaches according to the specific intervention’s beneficiaries, the products they use, and their modes of consumption (e.g., injection versus inhalation, smoking or ingestion).

With regard to the HR model as a whole, initiatives concerning drugs are very often financed by global health organizations; this poses difficulties for the sustainability of interventions not funded at the national level, a well-known problem in the field of programme development, and analysed by Olivier de Sardan [24]. This suggests the need to mobilize national funding for approaches and interventions that have a significant impact on the dynamics of drug use. In healthcare frameworks that still have a prohibitionist approach to drugs and in societies that morally condemn their use, HR programmes do not always correspond to political priorities. Accordingly, ‘imported’ HR programmes, such as ‘traveling models’ or ‘global templates’, should take into account social and cultural specificities, the diversity of user profiles and their social environment [18]. These initiatives cannot be considered *gender transformative*^13^ because they do not allow for long-term rehabilitation or empowerment of all PWUD.

The socioeconomic and professional reintegration of PWUD is one of the fundamental steps for improving their mental and physical health. Employment not only allows a person to regain status and a role in society, but also strengthens their self-esteem. In the case of WWUD, looking for a job is all the more difficult given that besides being drug users, they also face structural vulnerabilities linked to gender. These vulnerabilities include problems such as poverty, precariousness work and poor salaries which affect women in general, to which one can add stigmatization of women who may be physically marked by their years of consumption or who have a reputation associating them with the drugs and prostitution scene. In the Senegalese context, where the job market is very limited and dominated by the informal sector, it might be useful to have workshops on women’s employment, where women analyse and share their expectations in terms of employment, and are helped to find centres and services in their region that help in the professional reintegration process [25]. Any such workshop adapted to Senegal would have to make the link with social services and projects helping women. Well aware of these challenges facing HR stakeholders, the WWUD who created Femmes Engagées hope that the association will allow them to continue their search for help in social reintegration, something that is essential for populations suffering from an intersectionality of vulnerabilities.

In the field of addiction, a variety of therapeutic group models exist internationally including talk or focus groups, information and therapeutic education, (re)socialization, bodily mediation, memory and senses, artistic mediation with a creative or expressive aim, health, and justice. One of CEPIAD’s buildings in Dakar, which is still sometimes called the “Collomb’s hut”, was the location of the ‘penc’ (in Wolof) in the 1960s. These were therapeutic exchanges initiated by the French psychiatrist Henri Collomb who fostered the integration of traditional care by healers and the consideration of the cultural dimension of mental illness into routine hospital care. Indeed, at the end of the French colonial period, and in the first years of independent Senegal, the Dakar School laid the foundations of ethnopsychiatry through the use of therapeutic exchanges in the form of focus groups with patients, care providers and doctors [26–28]. This model was adapted to today’s needs and is currently being offered to women at CEPIAD.

Irrespective of the model used, our analysis shows that it should be adapted to the specific needs of WWUD in order to overcome the difficulty in mobilizing them. WWUD should be involved in the development and design of the objectives of any focus group and of “women-only” days in HR centres, as this would constitute an important determinant of the acceptability of the final chosen HR model [29]. The effectiveness of “traveling models” (see above) is recognized despite their limitations in terms of adaptability. They must be made more sensitive to the context so that they better take into account the constraints, resources, aspirations and strategies of local stakeholders [17].

Gynaecological follow-up is an important health need for WWUD. Some WWUD occasionally work as sex workers or temporarily engage in monetized sexual transactions. At the global level, women’s sexual and reproductive health includes standard gynaecological follow-up, the offer of contraception, additional gynaecological examinations, prenatal consultations, as well as screening for infectious comorbidities and cervical cancer [30]. For example, in France, in Addiction Care, Support and Prevention Centres (CSAPA) and in Reception and Support Centres for HR for drug users (CAARUD), most centres focus on “women” and on “mothers” offer perinatal monitoring, maternity support and help on parenting [30]. This ‘maternity’ and ‘mother-child bond’ dimension is not taken into account very much in the WWUD-specific offer at CEPIAD. The integration of permanent gynaecological services into care services for PWUD by strengthening collaborations between institutions, could allow WWUD to access not only better monitoring of sexual and reproductive health, but also to benefit from bio-medical-social care during pregnancy, something which would be helpful for both the mother and child.

In the field of addiction, the implementation of HR offers PWUD the opportunity to participate in public policy making. The model of self-support groups mostly promoted by community stakeholders in Senegal (e.g., ANCS, Enda Santé) has been recognized as effective in empowering peers in the PWUD rehabilitation process. This model is based on the fact that the failure of institutions and professionals to take into account the needs of patients can be compensated for by support between peers [31]. PWUD involved in associations are often implicated in discussions by local and international bodies regarding modalities of care. At the international level, self-support for PWUD generally concerns either “mutual aid” or “the defence of interests”. The former involves providing psychosocial support to PWUD, following the model of Alcoholics Anonymous (AA) born in the USA in the early 1950s. The latter consists in making social and political demands and promoting their expertise as PWUD. One example is the association ASUD in France, which campaigns for a reform of drug policies involving anti-prohibition, HR, and the rehabilitation of PWUD as full citizens through the fight against stigma and discrimination [31, 32].

With regard to Senegal, to our knowledge, no study to date has investigated PWUD association-based mobilization, or focused on women’s associations as a whole in the country by addressing the intersectionality of different types of mobilizations. Ndione (2018) [33] showed that the actions of the first associations set up in Senegal (APSUD^14^, ASRDR^15^) did not technically respond to the fundamental principle of an association - that is to say an interest group mobilizing for issues which concerns its members - because they presented themselves more as “mobilizations under guardianship” or “with a guardian”. In other words, they were driven by stakeholders in the field of intervention (e.g., ANCS) and health (in particular addiction psychiatrists) but not by the community members themselves [33]. Having said that, these associations have become more independent over the years, and today the demands they make differ from those that their ‘guardians’ initially put forward for them.

Since 2015, several self-support PWUD groups have been created in many regions of Senegal; the formalization of PWUD associations - such as SEV, ALT 2 S, SMS and more recently Femmes Engagées - is still supervised by the ANCS and Enda Santé. This suggests the value of conducting in-depth studies on (1) the community response to HR interventions, (2) the role of peer workers in HR, (3) the association-based dynamics of PWUD in Senegal, and especially (4) current mobilizations of WWUD, by examining their process of empowerment in future years.

## Conclusion

This study aimed to analyse the integration of gender into harm reduction initiatives in Senegal to better address the needs of women who use drugs. The findings reveal that the health and social care services established by these initiatives do not adopt a sufficiently gender-sensitive approach, leaving several categories of women (defined by their social context and the drugs they consume) outside their scope of action. The issues raised by this research are not limited to drug addiction or to a marginalised population. First, drug injection poses a risk for the transmission of infectious diseases such as HIV and hepatitis, hindering efforts to eliminate HIV in societies where prevalence is low in the general population but high among key populations, such as people who inject drugs. Second, the diversity of profiles highlights that invisibility particularly affects the consumption of medications and alcohol. Some female consumers remain “socially invisible,” such as those from affluent backgrounds who do not seek care and do not exhibit signs of social marginalization. This underscores the need to diversify responses and establish better coordination between regulation and care. Our study also highlights that the epidemic of addiction to medically prescribed tramadol in women in West Africa is growing (reflecting observations in North America). Finally, our work highlights gender differences in drug use and the specific needs of WWUD (across multiple categories) which the CEPIAD and various HR programmes in Senegal only partially address. HR services could be improved by incorporating our results. To address these challenges, it is essential to first develop harm reduction (HR) services tailored to the specific needs of women who use drugs (WWUD) who do not inject drugs. Additionally, further research is needed to better adapt interventions to this target population. Finally, mobilising funding is critical to implementing innovative and effective approaches that enhance their access to care.

## Data Availability

The data supporting the results of this study come from an anthropology thesis defended in 2022 at Cheikh Anta Diop University (UCAD). The manuscript is available from the library of Cheikh Anta Diop University, but restrictions apply to the availability of these data, which were used under license for the current study and are therefore not accessible to the public. The data are however available from the authors on reasonable request and with the permission of the principal investigators of the CODISOCS project, ANRS and IRD.

## Footnotes

1 The CODISEN project: Cohort of Users of Injectable Drugs in Senegal (ANRS 12334), and the CODISOCS project: Users of Injectable Drugs and Social Dynamics in Senegal (ANRS 12383), funded by the French National Agency for Research on AIDS and Emerging Infectious Diseases.

2 https://www.dedoose.com/. Qualitative data analysis software developed by academics at the University of California, Los Angeles.

3 The two projects received ethical and scientific approval from Senegal’s National Ethics Committee for Health Research and received administrative authorization from the Senegal Ministry of Health and Social Action, respectively, on 21 April, 2016 for CODISEN (notice no. 00000060 MSAS/DPRS/CNERS; extension number 00000163 MSAS/DPRS/CNERS 5 September, 2019), and 1 June 2018 for CODISOCS (notice no. SEN18/21 and approval no. 00000693 MSAS/DPRS/DR).

4 Outreach is based on the idea that waiting for drug users to come to care structures is not a viable option. Instead, it is grounded in the principle of “going to”, that is to say going to the places where drug users consume and providing care and support to them in situ, with a view to keeping them in good health. Peer education is an approach to health promotion that postulates that users and ex-users are the persons most able to have relevant discussions about harm reduction with other users, and to foster new HR practices in the latter. A drug use outreach team was set up in CEPIAD in 2009, and is one of the centre’s various care teams.

5 The three “key populations” in the field of HIV are PWUD (IDU), sex workers and men who have sex with men (https://www.unaids.org/fr/topic/key-populations ).

6 An international NGO whose objective is to improve prevention and access to quality care by strengthening the health systems and services of the countries where it operates.

7 A project implemented in Senegal, Mali and Ivory Coast by the NGO SOLTHIS; the project’s research dimension is supported by the French Institute of Research and Development (IRD); ATLAS focuses on the introduction of self-screening models in Africa. The project makes self-tests available to persons living with HIV and key populations through primary (i.e., direct to user) and secondary (i.e., using intermediaries) distribution. ATLAS aims to reduce morbidity and mortality from HIV/AIDS by improving access to HIV diagnosis and ensuring effective linkage to care.

8 This means “neighbourhood godmothers” in Wolof, the main language of Senegal. They are female community health workers who provide care, advice on sexuality and on sexual and reproductive health. They are often mobilised in local community health activities in Senegal.

9 *Association sénégalaise pour la prise en charge du VIH chez les usagers de drogues*. (Senegalese association for the treatment of HIV in drug users)

10 *Geum sa bopp* is a capacity building project for three PWUD associations. It has three components: capacity building, financial empowerment and the creation of a consultation framework between community stakeholders. Each association identified a collective IGA of its choice and received from support from Enda Santé (training, support for setting up the project). Two of these associations chose to set up chicken coops as their IGA.

11 intervention planning that includes specific actions to try to reduce inequalities between men and women within communities.

12 Gender blind: HR intervention planning that does not take into account gender differences and the specific needs of women, men, boys and girls, and which does not consider gender power dynamics, to the detriment of women and girls.

13 Gender Transformative: Intervention planning designed around a fundamental goal of addressing the root causes of inequality between men and women in society.

14 Medical initiative association in collaboration with the ANCS. It was set up by a UDI and a psychiatrist around 2005 and then dissolved in the face of constraints (financing, disagreement about the association’s objectives, etc.)

15 Senegalese association for the reduction of infectious risks among vulnerable groups. It was formed around an addictologist psychiatrist and a social worker after the 2011 UDSEN survey. Its objective is to improve the medical, social, economic and community care of CDIs in Dakar. It is aimed at key populations (IDU, PS, MSM).

## References

[1] Myers B, Carney T, Wechsberg WM. Not on the agenda: A qualitative study of influences on health services use among poor young women who use drugs in cape town, South Africa. Int J Drug Policy 1 Avr. 2016;30:52–8. 10.1016/j.drugpo.2015.12.019 PMC4829448 26797188

[2] Lambdin BH, Bruce RD, Chang O, Nyandindi C, Sabuni N, Zamudio-Haas S, et al. Identifying programmatic gaps: inequities in harm reduction service utilization among male and female drug users in Dar Es Salaam, Tanzania. PLoS ONE. 2013;8(6):e67062. 23825620 10.1371/journal.pone.0067062 PMC3692420

[3] Zamudio-Haas S, Mahenge B, Saleem H, Mbwambo J, Lambdin BH. Generating trust: programmatic strategies to reach women who inject drugs with harm reduction services in Dar Es Salaam, Tanzania. Int J Drug Policy 1 Avr. 2016;30:43–51. 10.1016/j.drugpo.2016.01.012 PMC4829444 26880500

[4] Mburu G, Ayon S, Tsai AC, Ndimbii J, Wang B, Strathdee S et al. Who has ever loved a drug addict? It’s a lie. They think a ‘teja’ is as bad person: multiple stigmas faced by women who inject drugs in coastal Kenya. Harm Reduct J. 25 mai. 2018;15(1):29. 10.1186/s12954-018-0235-9 PMC5970466 29801494

[5] Faye RA. Genre et addictions: les trajectoires des femmes usagéres de drogues Au Sénégal [Thèse de doctorat unique]. [Dakar. Sénégal]: Université Cheikh Anta Diop de Dakar. 2022. https://hal-emse.ccsd.cnrs.fr/ETHNO/tel-04952503v1

[6] Leprêtre A, Ba I, Lacombe K, Maynart M, Toufik A, Ndiaye O et al. Prevalence and behavioural risks for HIV and HCV infections in a population of drug users of Dakar, Senegal: the ANRS 12243 UDSEN study. Journal of the International AIDS Society [Internet]. 22 mai [cité 22 août 2017]. 2015;18(1). Disponible sur: http://www.jiasociety.org/index.php/jias/article/view/19888 10.7448/IAS.18.1.19888 PMC4442125 26004637

[7] Guillaume DBT, Chaka CO. Profils des femmes A consommation problematique de drogues En Côte D’Ivoire. Eur Sci J ESJ 31 Oct. 2017;13(29):306.

[8] Héritier F. Masculin/Féminin 2: dissoudre La hiérarchie. Paris: Odile Jacob. 2002. p. 443.

[9] Sow F. Genre et fondamentalismes/Gender and Fundamentalisms [CODESRIA [cité 18 mars 2021]. 2018. https://international.scholarvox.com/catalog/book/docid/88863834?searchterm=Fatou%20Sow

[10] Handman MÉ. Sexe Ou genre? Qu’en dit l’anthropologie sociale? Les nouvelles de l’archéologie. Genre et archéologie 30 Juin. 2015;(Number 140):5–8. https://journals.openedition.org/nda/2956

[11] Germes M, Künkel J, Langlois E, Perrin S, Scavo R. Espaces genrés des drogues. Parcours dans la fête, l’intimité et la réduction des risques [Internet]. Le Bord de l’eau. 2022 [cité 27 oct 2023]. 216 p. (Documents). Disponible sur: https://hal.science/hal-03669706

[12] Coppel A, Perrin S. Women, gender and drugs: between research and action. Harm Reduct J 15 Nov. 2024;21(1):200. 10.1186/s12954-024-01106-7 PMC11566170 39548469

[13] Percival V, Dusabe-Richards E, Wurie H, Namakula J, Ssali S, Theobald S. Are health systems interventions gender blind? examining health system reconstruction in conflict affected states. Globalization and Health. 30 août. 2018;14(1):90. 10.1186/s12992-018-0401-6 PMC6116483 30157887

[14] Mutatayi C, Morton S, Robles Soto N, Palsdottir KI, Vale Pires C, Pompidou Group. Implementing A Gender Approach in Drug Policies: Prevention, Treatment and Criminal Justice. Strasbourg, France: Council of Europe. 2022. 168 p. Available from: https://rm.coe.int/2022-ppg-implementing-a-gender-approach-in-drug-policies-a-pg-handbook/1680a66835

[15] Covington SS. Women and addiction: a trauma-informed approach. J Psychoact Drugs Nov. 2008;Suppl 5:377–85. 10.1080/02791072.2008.10400665 19248395

[16] Gaudilliere JP, Andrew McDowell, Lang C, Beaudevin C. Global health for all: knowledge, politics, and practices. New Brunswick, N.J: Rutgers University Press. 2022.

[17] Olivier de Sardan JP, Diarra A, Moha M. Travelling models and the challenge of pragmatic contexts and practical norms: the case of maternal health. Health Res Policy Sys Juill. 2017;15(S1):60. 10.1186/s12961-017-0213-9 PMC5516842 28722553

[18] Magen C. Pour quelle Réduction des Risques en Afrique et ailleurs? Observatoire de la santé mondiale. 2019;11.

[19] Desclaux A, Msellati P, Sow K. Les femmes à L’épreuve du VIH Dans les pays du Sud. Genre et accès universel à La prise En charge. Paris: ANRS. 2011. p. 256. (Sciences sociales et sida).

[20] Stengel CM, Mane F, Guise A, Pouye M, Sigrist M, Rhodes T. They accept me, because I was one of them: formative qualitative research supporting the feasibility of peer-led outreach for people who use drugs in Dakar, Senegal. Harm Reduction Journal. 27 févr. 2018;15:9. 10.1186/s12954-018-0214-1 PMC5830063 29486774

[21] Felicien Yomi T, Tokou Antoine A, Dié N’dri Sandrine K, Matina K, Edith A, Désiré Déjourneux A. Accès aux soins des femmes usagères de drogues précaires à Abidjan (Côte d’Ivoire): l’expérience de l’Association communautaire Paroles Autour de la Santé [Internet]. 2023 [cité 18 nov 2024]. Disponible sur: https://inhsu.org/resource/acces-aux-soins-des-femmes-usageres-de-drogues-precaires-a-abidjan-cote-divoire-lexperience-de-lassociation-communautaire-paroles-autour-de-la-sante/

[22] Ayon S, Ndimbii J, Jeneby F, Abdulrahman T, Mlewa O, Wang B et al. Barriers and facilitators of access to HIV, harm reduction and sexual and reproductive health services by women who inject drugs: role of community-based outreach and drop-in centers. AIDS Care. 3 avr. 2018;30(4):480–7. 10.1080/09540121.2017.1394965 29067855

[23] Desclaux A, Desclaux Sall C, Sow K. In. Un Seul modèle pour Tous? De La diversité des modes d’engagement communautaire Dans La recherche à Dakar, Sénégal. in La recherche communautaire VIH/sida: Des savoirs engagés. 2015. pp. 212–20. Santé et société, Presses de l'Université du Québec. https://www.jstor.org/stable/j.ctt1f1165j

[24] Olivier de Sardan JP. Anthropologie et développement: Essai En socio-anthropologie du changement social. Marseille: APAD [u.a.]. 1995. p. 221. (Hommes et sociétés).

[25] Rodriguez GH. The gender perspective in drug addiction programs and services. Dianova [Internet]. 2019; Available from: https://www.dianova.org/wp-content/uploads/2019/12/201911-la-perspectiva-de-genero-en-los-programas-Giseal-Rodriguez-Hansen-EN.pdf

[26] Boussat M, Boussat S. De La psychiatrie coloniale à Une psychiatrie Sans frontières. A Propos de Henri collomb (1913–1979). L’autre - Cliniques Cultures Et Sociétés. 2002;3(3):409–24.

[27] Boussat S, Boussat M. À Propos de Henri collomb (1913–1979): de La psychiatrie coloniale à Une psychiatrie Sans frontières. L’Autre. 2002;3(3):409–24.

[28] Collignon R. Henri collomb and the emergence of a psychiatry open to otherness through interdisciplinary dialogue in post-independence Dakar. Hist Psychiatry 1 Sept. 2018;29(3):350–62. 10.1177/0957154X18777210 29860874

[29] Charlier É. Groupes de parole Non thérapeutiques pour Proches: expériences vécues des acteurs. Approchesind. 2018;5(2):11–40.

[30] Mutatayi C. Résultats de l’enquête Ad-femina - Tendances 130 - mars 2019 - OFDT [Internet]. 2019 [cité 11 févr 2021]. Disponible sur: https://www.ofdt.fr/publications/collections/periodiques/lettre-tendances/resultats-de-lenquete-ad-femina-tendances-130-mars-2019/

[31] - Dekkers A, Vos S, Vanderplasschen W. Personal recovery depends on NA unity: an exploratory study on recovery-supportive elements in Narcotics Anonymous Flanders. 2020. available from: https://pmc.ncbi.nlm.nih.gov/articles/PMC7393873/10.1186/s13011-020-00296-0 PMC7393873 32736568

[32] Chappard P, Couteron JP, Sovape A, Chapitre D. 2019 [cité 31 mai 2021]. Disponible sur: https://www.cairn.info/addictologie-2019--9782100788408-page-585.htm

[33] Ndione AG. Les associations d’usagers de drogues injectables Au Sénégal: D’une participation Sous Tutelle à Un projet Autonome. Se mobiliser Contre Le sida En afrique Sous La Santé globale les luttes associatives. Dir. Christophe Broqua. L’Harmattan. Paris; 2018. (Anthropologie et médecine). https://imaf.cnrs.fr/IMG/pdf/se_mobiliser_contre_le_sida_en_af.pdf

